# Health seeking behavior of street connected children in Addis Ababa, Ethiopia

**DOI:** 10.3389/fsoc.2023.1188746

**Published:** 2023-08-07

**Authors:** Bewunetu Zewude, Getahun Siraw, Kibur Engdawork, Getnet Tadele

**Affiliations:** ^1^Department of Sociology, College of Social Science and Humanities, Wolaita Sodo University, Sodo, Ethiopia; ^2^Department of Sociology, College of Social Sciences and Humanities, Dilla University, Dilla, Ethiopia; ^3^Department of Sociology, College of Social Science, Addis Ababa University, Addis Ababa, Ethiopia

**Keywords:** street children, risk perception, prevention, illness response, susceptibility

## Abstract

**Background:**

Street children are the most neglected segments of the society with limited access to healthcare services. The vulnerability of street children to various health risks has been found by previous studies but little is known about their perceived susceptibility, preventive behavior and illness responses. Hence, the purpose of this study was to identify the health seeking behavior of street children in Addis Ababa. The study focuses on perceived susceptibility to various health risks, sources of health risks, and behaviors pertaining to responding to perceived risks and experienced health problems among the most marginalized groups in Addis Ababa.

**Methods:**

Using a mixed research approach, quantitative and qualitative data were collected through survey and interview methods from selected street children. SPSS and NVivo software were used to analyze the quantitative and qualitative data, respectively.

**Results:**

Whereas the street children perceive to be susceptible for ill-health risks related with their living situations, responding to the perceived susceptibility mainly by maintaining personal hygiene and undertaking physical exercises have been identified. The study also revealed that street children were found to be vulnerable for the situations affecting their health and wellbeing mainly due to self-reported engagements in risky behaviors such as smoking cigarette (67.3%), sniffing glue or benzene (68.2%), sharing of personal materials having the potential of transmitting diseases from one person to another (25.5%), and unprotected sexual activities (14.1%). Experiences of visiting healthcare facilities in response to illness symptoms have also constituted an aspect of the health seeking behavior of the street children.

**Conclusion:**

Awareness of the presence of health risks and perceived susceptibility to the risks promoted both preventive behavior and positive compliance in relation to illness response among children of the street in Addis Ababa.

## Introduction

Health-seeking behavior refers to any action undertaken by individuals who perceive themselves to be susceptible to or have faced health problems for the purpose of finding an appropriate remedy and promoting good health status (Oberoi et al., 2016; Mushtaq et al., 2020). Cornally and McCarthy (2011) defined it as a problem-focused, planned behavior involving interpersonal interaction with a selected healthcare professional. Moreover, it is situated within the broader concept of health behavior that encompasses a range of activities undertaken to maintain good health, prevent negative health outcomes, and actions taken to restore a good state of health (Latunji and Akinyemi, 2018). Health-seeking behavior passes through a logical sequence of steps that begin with the perception and evaluation of symptoms and end with the utilization of different healthcare services (Mackian, 2004). It includes all the behaviors undertaken to maintain a healthy physical and mental state (primary prevention), to restore any deviation from normal health conditions (secondary prevention), and to reduce the impacts and progression of an illness (tertiary prevention) (Uche, 2017). According to Latunji and Akinyemi (2018), the state of the health-seeking behavior of members of a given society can be an important indicator of society's well-being and is linked to its overall socio-economic development. Above all, people's responses to symptoms of illness have significant implications for morbidity and progression of the illness. In other words, delaying or refusing to seek and contain a proper diagnosis and medical treatment are more likely to cause adverse negative health consequences (Afolabi et al., 2013; Adam and Aigbokhaode, 2018).

The nature of health-seeking behavior varies from person to person or between groups depending on different factors (Oberoi et al., 2016) which tends to result in varied levels of consumption of healthcare services between individuals in a group or across different groups in a society. According to Mackian (2004), the determinants of health-seeking behavior can broadly fall under geographical, social, cultural, economic, and organizational factors. Siddiqui (2014) identified factors such as the status of women, age, sex, household resources, costs of care, distance and physical access to healthcare services, perceived quality of healthcare services, and standards of drugs affecting health-seeking behavior. In addition, Basharat et al. (2019) stated that lack of awareness about risk factors, symptoms, and approach to treatment were the main reasons for delaying treatment. A study on the health-seeking behavior of slum dwellers in Dhaka City, Bangladesh (Jahan et al., 2015) found that limited access to healthcare is a serious constraint on the use of healthcare services. Likewise, Adane et al. (2017) concluded that increasing the proximity of health facilities, health education, and socio-development programs targeting illiterate mothers/caregivers and poor households may promote and increase health-seeking behavior in slum areas in Addis Ababa City.

Other studies have found factors such as the amount of social capital, level of access to health information, availability of providers, communication barriers, the need to keep one's health problem secret, availability of opportunities for specialized care, fear of side effects of drugs, level of education, type and severity of illness, costs of care in relation to time, travel and treatment, attitudes, beliefs and core values, life adaptation skills, psychological dispositions, social support, media, exposure to health information, gendered preference of care providers, religion, culture, marital status, income, occupation, work time of healthcare facilities, and the nature of the healthcare environment such as long waiting time, misbehavior of staffs, negligence of doctors, willingness to listen to disease history, commercial attitudes to prescribe pathological tests affecting health seeking behavior (Mackian, 2002; Afolabi et al., 2013; Webair and Bin-Gouth, 2013; Tegegne and Legese, 2014; Akeju et al., 2016; Rahman et al., 2016; Uche, 2017; Adam and Aigbokhaode, 2018; Latunji and Akinyemi, 2018; Basharat et al., 2019; Jalu et al., 2019; Khalil et al., 2019; Guta et al., 2021). According to Mackian (2002), health-seeking behavior is not just an isolated event; rather, it is the result of an evolving mix of social, personal, cultural, and experiential factors.

Being a street child involves vulnerability to multiple grounds of disadvantageous positions, mainly as far as health and well-being are concerned (Chowdhury et al., 2017; Tahmina et al., 2018; Said and Aldewachi, 2020; Abate et al., 2022). A review of the literature on the health conditions of street children in selected African countries (Cumber and Tsoka-Gwegweni, 2015) revealed that street children are vulnerable to poor health conditions, including violence, injuries, and HIV/AIDS, mainly because of factors such as homelessness, risky sexual behavior, and substance abuse. According to Ali and de Muynck (2005), street children are highly susceptible to adverse health outcomes such as physical injuries and respiratory and skin infections. Furthermore, a study conducted in Northern Ethiopia (Brhane et al., 2014) revealed that one-third of street children had started sexual intercourse, over 60% of them had more than one sexual partner, and 40.6% had sexual intercourse with commercial sex workers. Above all, Eshita (2018) concluded that street children are the most neglected part of society, with very negligible access to health care and lack of awareness of available health services.

Identifying the prevalence and extent of differences in health-seeking behavior across various social groups is crucial because of its implication on inequality in the access and utilization of healthcare facilities among members of diverse social groups. Besides the susceptibility of street children to various ill-health conditions, it is important that their health-seeking behavior be understood due to its policy implications for healthcare development and the reintegration of such marginalized social groups (Afolabi et al., 2013; Uche, 2017). Moreover, the health-seeking behavior of street youth in Ethiopia has not been adequately studied, and our knowledge in this case is mostly limited to the higher susceptibility to ill-health conditions among members of the social group (e.g., Tadele, 2009; Kapali, 2011; Brhane et al., 2014; Zewude, 2018; Abate et al., 2022). Therefore, the present study considered health-seeking behavior within the broader context of health behavior, including perceived susceptibility to health risks, preventive behavior against risks, patterns of illness responses, alternative care-seeking behavior, and health-related decision-making processes among street children in Addis Ababa, Ethiopia.

## Materials and methods

### Study area

The study was conducted in Addis Ababa, Ethiopia. Compared to other parts of the country, the issue of streetism has become a grave concern in Addis Ababa (Bekele, 2020). According to a scoping study of MOLSA (2018), Addis Ababa and DireDawa take the lion's share as far as the distribution of urban destitute living on street sides of Ethiopia is concerned. With more than 3,384,569 million dwellers according to the 2007 census and the 2022 projected population size of 5, 227,794 (with 4.44 growth rate), Addis Ababa is by far the largest city and is home to about a quarter of the country's urban population. Most of the development is concentrated in the city and it has always been experiencing large influx of people from rural areas and other smaller towns. The city is characterized by fast changing situations having a potential consequence of increasing the risk of involvement into street life for large number of vulnerable people living in the city, especially the urban poor (Veale et al., 1993). Conducting the study in Addis Ababa is necessary due to high prevalence of streetism (MOLSA, 2018; StreetInvest, 2020) and its consequences, such as high crime rates and decreasing environmental sanitation.

### Research design

Conticini and Hulme (2007) suggest that the use of statistical data alone is less helpful for researches investigating children in street situations. “Every child has a unique story to tell. As important as it is to quantify this phenomenon, numbers are of little help in understanding the context in which they live, the desperation that leads them to run away from home, and the challenges they struggle with to survive on the streets” (Ferrara and Ferrara, 2005, p. 1). Accordingly, the study combined both qualitative and quantitative methods to identify the health seeking behavior of street children in Addis Ababa. We used a concurrent nested research design where methods and data were triangulated in addressing one or more research objectives (Barnes, 2019). Whereas survey was used to gather the quantitative data, interview method has been used to collect qualitative data from selected street children. According to Brierley (2017), a mixed methods design is useful to get a complete picture of the research question and a full understanding of what is being done.

### Research methods and data sources

A cross-sectional survey was conducted in four sub-cities of the Addis Ababa City Administration in Ethiopia. The survey was pilot-tested with 20 street children to assess the adequacy of the instruments. The results of the pilot test showed that some questions were not clear for the street youth, which resulted in respondents requesting clarification. Accordingly, the researchers revised the instruments. After receiving feedback and making all the necessary corrections, the questionnaires proportional to the calculated sample size were duplicated. In addition, for the purpose of triangulating data, qualitative data were collected from research participants using in-depth interview method. Semi-structured questions were developed to guide the interview sessions.

### Sampling and selection of research participants

The main methodological constraint encountered when studying street children is the impossibility of adopting sampling procedures to ensure that the relatively small group of interviewees represents the composition of the larger population of children in street situations (Conticini and Hulme, 2007). A one-stage cluster sampling technique was used to select the participants. Given the highly mobile nature of the study population, finding an appropriate population size concentrated in the study area was impossible, which makes obtaining sampling frames difficult. Therefore, research participants were randomly contacted to collect data through an interviewer-administered questionnaire. Accordingly, the major inclusion criteria were the age of respondents being within the category of 10–17, being children of the street who use the street both as a shelter and source of livelihood, and willingness to participate in the survey. The period of data collection coinciding with the rainy season in Ethiopia (end of May 2022 and June, 2022) the highly mobile nature of the study population reduced the predictability of their location, and the range of time suitable to interview the children being very limited, data collectors were able to contact 230 street children from which 220 cases were found to be completed and clean to be inserted in SPSS software. For the qualitative approach, a purposive sampling technique was used in which the researchers tried to maintain maximum variation in the characteristics of the chosen samples to include diverse perspectives and insights into the research. In addition, the sample size was the number of interviewees counted until the point at which a state of data saturation was attained in the sense that the researchers realized no new information was discovered, and enough data were collected to replicate the study (Fusch and Ness, 2015). Accordingly, 22 street children were interviewed in this study.

### Instrument design

The questionnaire used to gather quantitative data for the present study was developed on the basis of Babitsch et al. (2012) and after an exhaustive review of previously published relevant research articles. The outcome (dependent) variable is the health-seeking behavior of respondents, measured in terms of both preventive health behavior and illness response behavior, whereas the independent variables include socio-demographic characteristics of the respondents, such as age, sex, education, religion, residential background, and the number of years spent on the street. The questionnaire mainly consisted of three sections: a section asking about the socio-demographic characteristics of respondents, the second section focusing on primary prevention behavior, and the third section related to respondents' illness response behavior. Accordingly, the first section seeking the socio-demographic characteristics of survey participants included questions such as age, sex, religion, educational status, residential background, and the number of years they stayed on the street. The second section consisted of questions regarding the respondents' primary preventive behavior. There were (1) “is there anything you do to protect yourself from ill-health conditions?” (2) “Have you ever had unprotected sexual intercourse/no condom use?” (3) “Do you maintain your personal hygiene (taking shower, changing cloths, etc)?”, and (4) “Have you ever visited healthcare facilities (hospital/clinic/health center) for general health check-up?” among others. Finally, the third section of the questionnaire aimed to measure the illness response behavior of respondents and included questions such as (1) “Have you ever been sick since you joined street life?” (2) “If yes, have you ever visited healthcare facilities for diagnosis/treatment?”, and (3) “What do you commonly do whenever you feel sick?”, among other questions.

### Method of data analysis

First, the data were checked for completeness. Only the questionnaires that were found to be correctly filled were inserted into SPSS software version 26. Data analysis was conducted using statistical techniques, including percentage and frequency distributions. Descriptive statistical techniques were used to analyze and present issues such as the socio-demographic characteristics of respondents, the situation of health risk perception, related preventive behavior, and patterns of illness responses among the respondents, among other things. For qualitative data, the audio records were first transcribed verbatim and then translated into English. The data were then coded using NVivo version 12 software followed by deep reading and identification of themes and sub-themes. Subsequently, the data were interpreted in relation to the specific research questions.

### Data quality management

According to Costa (2022), “good data” are characterized mainly by accuracy, relevance, completeness, timeliness, and consistency. Because data quality management is a continuous process it was undertaken in three phases: before the field work, during data collection, and after the field during data entry and analysis. Before the field, all prescribed rules and principles were followed during questionnaire construction and the development of all other data gathering tools. Both face and criterion validity of the instruments were established, such as carefully selecting the variables/items after exhaustive review of the literature (Heale and Twycross, 2015). As much as possible, the process of recruiting data collectors considered previous exposure to street connected children or experience of undertaking relevant researches and implementation of community-based project works. In addition, sufficient training was given to data collectors with a strong supervision and field coordination made by the researcher. Pilot testing of the data collection instruments with relatively small number of street children has also been undertaken. Moreover, data were carefully entered into the software and stored on computer with a separate folder created for this purpose. Similar data quality assurance and controlling procedures were followed during data analysis and interpretation.

### Ethical considerations

The characteristics of the study participants being under age and having low level of literacy makes it difficult for them to provide informed consent to participate in the study. Therefore, the researchers used research approaches that involve as minimum risk as possible to the safety and privacy of the children. According to Kaime-Atterhog (2012), allowing the street children to lead the research and having a long and sustained period of engagement in the field ensures that the interests of the children gets priority. Children were provided with adequate information, among other things, about what the research is all about, the level of potential risks involved as a result of participating in the research, the type of information required from them, and that the information they provide will be kept confidential. Maximum effort was also made to make sure that no child has been put into a risk as a result of participating in the study. In this regard, the fundamental principles of “do no harm” and “taking into account the best interest of the child” were kept throughout data collection and presentation of data. Above all, because consent is an ongoing process (Smart, 2018), children have been assured and reassured to withdraw from the research any time they feel discomfort, by telling them that such withdrawal does not involve any negative consequences to them.

## Results

The socio-demographic characteristics of the participants are presented in Table 1. Accordingly, it is found that most (90.9%) of the respondents are males and the average age of the participants has been 15.27 (SD: 1.42). In addition, their educational background revealed that the majority (83.6%) had attended only the primary level of education. Furthermore, while half (50%) of the respondents reported being Orthodox Christians in their religious faith, most (60.9%) replied that they had been raised in urban areas. Data regarding the period counted since joining street life revealed that most (43.2%) had stayed 1–3 years on the street.

**Table 1:** The socio-demographic characteristics of respondents.

**Variables**	**Categories**	**Frequency (%)**
Sex	Male	200 (90.9%)
	Female	20 (9.1%)
Educational status	Never attended school	26 (11.8%)
	Primary level (1–8)	184 (83.6%)
	Secondary level (9–12)	10 (4.5%)
Religious background	Orthodox Christian	110 (50%)
	Muslim	74 (33.6%)
	Protestant	30 (13.6%)
	Catholic	4 (1.8%)
	Adventist	1 (0.5%)
	Atheist	1 (0.5%)
Duration on the street	< 1 year	48 (21.8%)
	1–3 years	95 (43.2%)
	4–6 years	64 (29.1%)
	7–9 years	13 (5.9%)
Residential background	Urban	134 (60.9%)
	Rural	86 (39.1%)
Total		220 (100%)

### Primary prevention behavior: perceived susceptibility and actions taken to prevent health risks

As shown in Table 2, most respondents (71.4%) perceived that they were susceptible to health risks as a result of their living conditions on the street. In addition, data have shown that street children are vulnerable to situations affecting their health and wellbeing, mainly due to self-reported engagement in risky behaviors. For instance, it was found that 67.3% of respondents smoked cigarettes, 68.2% had sniff glue or benzene, 25.5% shared personal materials with the potential to transmit diseases from one person to another, and 14.1% had unsafe sexual intercourse. Moreover, the results showed that three-quarters of street children had never visited health care centers. The absence of experience visiting healthcare facilities for the purpose of undergoing a general health check-up among most respondents (75.5%).

**Table 2:** Frequency distribution of perceived susceptibility and preventive behavior of respondents.

**Variables/questions**	**Categories**	**Frequency (%)**
Have you ever been concerned about being infected by a disease while living on the street?	Yes	157 (71.4%)
	No	63 (28.6%)
Have you ever had unprotected sexual intercourse/without the use of condom?	Yes	31 (14.1%)
	No	189 (85.9%)
Have you ever been tested for sexually transmitted diseases such as HIV/AIDS?	Yes	10 (4.5%)
	No	21 (9.5%)
Do you maintain your personal hygiene (taking shower, changing cloths, etc)?	Yes	199 (90.5%)
	No	21 (9.5%)
Do you smoke cigarette?	Yes	148 (67.3%)
	No	72 (32.7%)
Do you sniff glue/benzene?	Yes	150 (68.2%)
	No	70 (31.8%)
Do you share personal materials such as blade, needle, and teeth brush with your friends?	Yes	56 (25.5%)
	No	164 (74.5%)
Have you ever been using marijuana since you joined street life?	Yes	61 (27.7%)
	No	159 (72.3%)
Have you ever visited healthcare facilities for general health checkups?	Yes	54 (24.5%)
	No	166 (75.5%)
Total		220 (100%)

**Figure 1:**
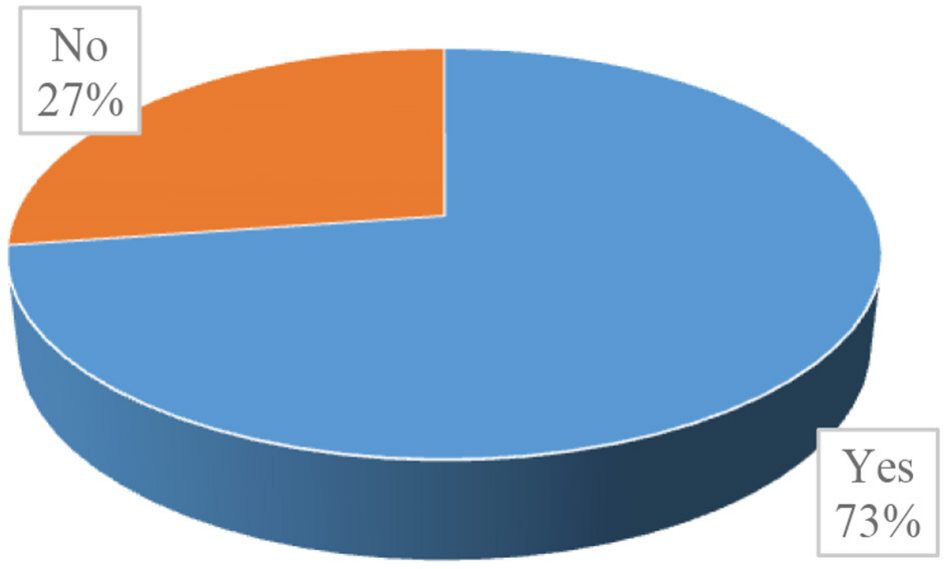
Is there anything you do to protect yourself from ill-health conditions?

Respondents were asked whether there was something they did in response to the perceived or experienced health risks. According to the data presented in Figure 1, 73% of respondents replied that they do something to protect themselves from ill-health conditions, implying the existence of preventive health behaviors among street children in the study area. In addition, practices that positively contribute to the health and well-being of the respondents, such as experiences of maintaining personal hygiene (90.5%) with a relatively high interval of taking a shower, have also been reported, as shown in Table 2.

The percentage distributions of health beliefs and subsequent preventive behaviors of respondents presented in Table 3 reveal that stomach ache (45.2%), louse-borne diseases (38.2%), headache (36.9%), illnesses caused by exposure to harsh weather (36.3%), typhoid/typhus (22.3%), and cardiac infection (21%) were the health problems that respondents were most concerned about. In addition, maintaining personal hygiene (46.6%), doing physical exercise (44.1%), and prayer or spiritual activities (29.8%) were found to be the commonly used mechanisms used by respondents to protect themselves from ill-health conditions. On the other hand, for the respondents who disclosed that they do not do anything to protect themselves from health risks (27%, as shown in Figure 1), the most frequently reported reasons or barriers were financial problems (66.7%), their housing situation (23.3%), absence of health-related knowledge (16.7%), and absence of facilities (15%).

**Table 3:** Health beliefs and related preventive behavior of respondents.

**Variables/items**	**Categories of responses**	**Responses**		**Percent of cases**
		***N***	**Percent**	
The disease/ill-health conditions that respondents are most concerned about	HIV/AIDS	21	3.8%	13.4%
	Skin/dermal disease	20	3.6%	12.7%
	Other sexually transmitted infections	6	1.1%	3.8%
	Vector/louse borne diseases	60	10.9%	38.2%
	Heart Disease	33	6.0%	21.0%
	TB	13	2.4%	8.3%
	Typhoid/typhus	35	6.4%	22.3%
	Kidney failure	7	1.3%	4.5%
	Depression	25	4.5%	15.9%
	Stress	30	5.4%	19.1%
	Cancer	16	2.9%	10.2%
	Hepatitis	4	0.7%	2.5%
	Stomach-ache	71	12.9%	45.2%
	Headache	58	10.5%	36.9%
	Physical impairments	36	6.5%	22.9%
	Other accident-related injuries	19	3.4%	12.1%
	Illness caused by exposure to harsh weather	57	10.3%	36.3%
	Malaria	24	4.4%	15.3%
	Others	16	2.9%	10.2%
Total		551	100.0%	351.0%
Mechanisms of protecting oneself from ill-health conditions	Doing physical exercises	71	24.8%	44.1%
	Health check-ups	8	2.8%	5.0%
	Early reporting of symptoms	12	4.2%	7.5%
	Eating balanced diet	26	9.1%	16.1%
	Not using harmful drugs/substances	38	13.3%	23.6%
	Vaccination	7	2.4%	4.3%
	Prayer/other spiritual activities	48	16.8%	29.8%
	Maintaining personal hygiene	75	26.2%	46.6%
	Others	1	0.3%	0.6%
Total		286	100.0%	177.6%
Reasons for not doing something to protect one's health	Feelings of worthlessness	10	9.4%	16.7%
	Negligence due to running to meet survival needs	9	8.5%	15.0%
	Financial problems	40	37.7%	66.7%
	My housing situation	14	13.2%	23.3%
	Lack of health-related knowledge	10	9.4%	16.7%
	Absence of facilities	9	8.5%	15.0%
	Lack of access to healthcare services	6	5.7%	10.0%
	I feel that I should withstand challenges	1	0.9%	1.7%
	No reason	5	4.7%	8.3%
	Others	2	1.9%	3.3%
Total		106	100.0%	176.7%

### Illness response behavior of the respondents

**Figure 2:**
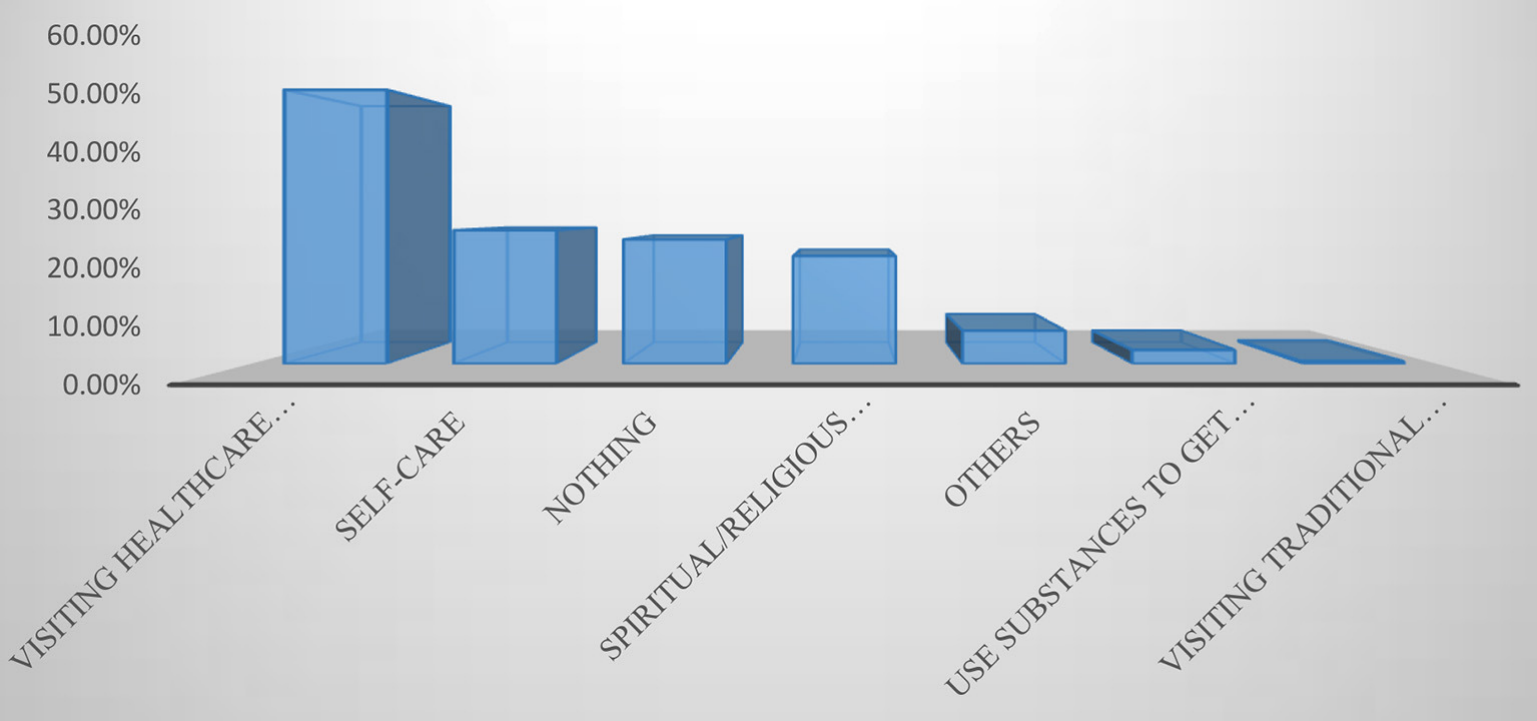
What do you commonly do whenever you feel sick?

Figure 2 shows that just over half of the street children reported visiting modern healthcare facilities whenever they felt sick, followed by self-care (25.9%), doing nothing (24.1%), and spiritual or religious treatment (20.9%).

**Figure 3:**
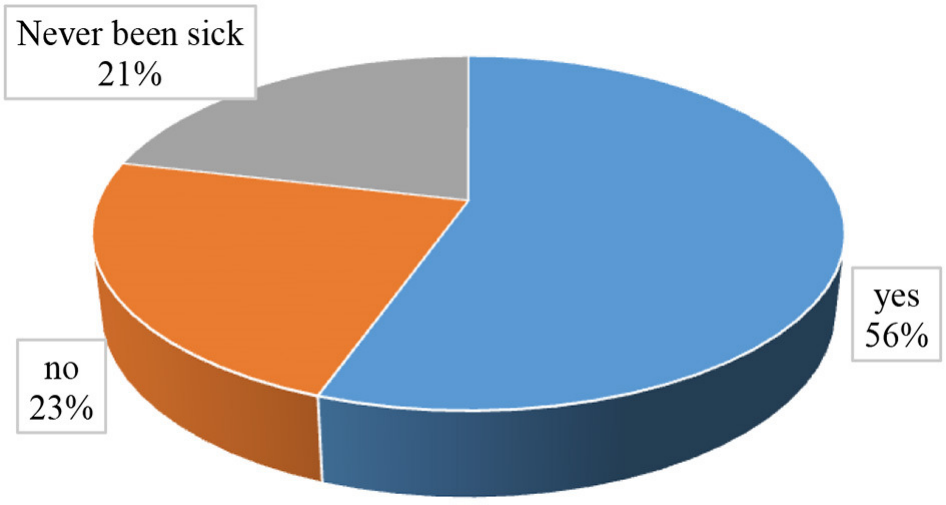
Have you ever visited healthcare facilities for diagnosis/treatment?

As presented in Figure 3, the percentage distribution of responses regarding respondents' experiences of visiting healthcare facilities revealed that most (56%) respondents had ever visited healthcare facilities.

Among the respondents who reported that they had never visited healthcare facilities (23%), as shown in Figure 3, the most frequently mentioned reasons were financial problems (64.7%), the disease being less severe (29.4%), preference for self-care (7.8%), fear of pain during medical injection (3.9%), fear of long waiting hours in healthcare facilities (3.9%), and lack of awareness (3.9%), as shown in Table 4. Furthermore, respondents were asked about the conditions that enabled them to visit healthcare facilities, and most (48.2%) replied perceived severity of the disease, followed by feeling ill (27.7%), the presence of an adequate amount of money (27.3%), peer pressure (24.5%), and access to free healthcare services (23.2%).

**Table 4:** Reasons for not seeking healthcare and conditions of seeking treatment.

**Questions**	**Categories of responses**	**Responses**		**Percent of cases**
		***N***	**Percent**	
What was your reason for not been visiting healthcare facilities for diagnosis/treatment?	The disease was not that severe	15	21.7%	29.4%
	Financial problem	33	47.8%	64.7%
	Use substances to get temporary relief	1	1.4%	2.0%
	Fear of pain during injection	2	2.9%	3.9%
	Didn't know I should visit health facilities	2	2.9%	3.9%
	I prefer self-care	4	5.8%	7.8%
	I prefer traditional medicine	1	1.4%	2.0%
	I prefer spiritual healing	1	1.4%	2.0%
	Distance of health facilities	3	4.3%	5.9%
	Communication problems with health workers	1	1.4%	2.0%
	Fear of long waiting time in the health facilities	2	2.9%	3.9%
	I believe that I should withstand challenges	1	1.4%	2.0%
	Others	3	4.3%	5.9%
Total		69	100.0%	135.3%
If you get sick, under which of the following conditions do you visit healthcare facilities?	I will never visit healthcare facilities	3	0.9%	1.4%
	As soon as I feel ill	61	17.8%	27.7%
	If I perceive that the disease is severe/deadly	106	31.0%	48.2%
	When other alternative trials fail	4	1.2%	1.8%
	When friends/others insist me to do so	54	15.8%	24.5%
	When I have adequate money	60	17.5%	27.3%
	When I get free healthcare services	51	14.9%	23.2%
	Others	3	0.9%	1.4%
Total		342	100.0%	155.5%

### Results from qualitative data: perceived susceptibility and responding to health risks

The study showed that street children perceive themselves to be highly susceptible to various health risks that affect their health and well-being. And one of the most frequently reported perceived health risks was “RF” which they believe is caused by the lack of personal hygiene.

*I am more afraid of RF*^1^ *because I have had previous experience with it. It is caused by a lack of personal hygiene and a sleeping area. RF is caused by having so many louses on your hair and clothes, especially if you don't wash it on a regular basis. Bed sheets we use to sleep on the ground may develop louse, especially if not washed on a regular basis. I was severely ill as a child as a result of this because I had no experience washing my body and clothes until I was older (XX7, male, 16 years old)*.

Others have expressed their concerns about the possibility of contracting lung cancer and kidney infection as a result of smoking cigarettes:

*Kidney disease and lung cancer. I'm especially concerned about lung cancer because a small wound on the lung can grow larger if you smoke cigarettes all the time. I am afraid of being exposed to cancer and becoming physically disabled as a result of having parts of my body cut to stop its spread (XX2, male, 15)*.

For another child on the street, kidney infection is perceived to be caused by stressful situations and the absence of care.

*It could be as a result of stress or from sleeping in cold weather. We also don't get up to urinate at night once we've fallen asleep because we usually sleep abnormally, possibly on top of others, and because we're afraid of the cold air condition. This may contribute to kidney problems (XX2, male, 15)*.

Interviewees disclosed that they are highly vulnerable to various sexually transmitted infections, especially HIV/AIDS, because of their risky sexual practices. For instance, a male adolescent of the street disclosed that he often engages in unsafe sex with his female counterparts on the street and even suspects himself to be infected by HIV/AIDS.

*Of all other diseases, I'm highly concerned about HIV AIDS because there are many females who live on the street like us. They come here, and we sometimes sleep together. Although we know the fact that some of them are already HIV positive, we boys living on the street don't use condoms. As a result, we are most likely to contract the virus from them*.

For most children on the street, susceptibility to ill-health conditions is believed to be associated with their living situation on the street:

*Living on the streets is a disease by itself. One would be susceptible to communicable diseases such as typhus and typhoid while living here (XX4, male, 17 years old)*.

*I think I am very much susceptible to disease due to my street sleeping situation on the street. I sleep on the street without a blanket and hence, vulnerable to illness as a result of the cold weather. I also believe that having louse infestations on my clothes would put me at risk of developing other health issues. For example, I usually encounter shortage of breath after running a very short distance (XX10, male, 14 years old)*.

*For one thing, I beg money from strangers, eat hotel leftovers, buy and chew khat, buy and drink local whisky or “Areke,” then get drunk and sleep on the ground, and so on. Because this is my daily routine, I am constantly exposed to various health problems (XX3, male, 18)*.

The harsh relationship between street children and the police in the city has been found to be another source of health risk for street children:

*We are concerned that the police's brutality will cause us to accidentally fall while running, putting us in danger of physical injury or in danger of being involved in a car accident (XX9, male, 14 years old)*.

*The police, however, intimidate us all to leave the area. They dislike seeing us sitting and having fun together. They also came unexpectedly late at night while we were sleeping, beat us severely, and forced us to flee to other locations at night (XX3, 18, male)*.

The study also indicated that perceived susceptibility to health risks is linked to the preventive health behavior of street children. As revealed by the participants, street children respond to perceived susceptibility using different mechanisms, where maintaining personal hygiene is a common practice:

*If I don't maintain my personal hygiene, I'm sure I would have been exposed to various diseases, especially RF. This is because RF is caused by having so many louses on your hair and clothes, especially if you don't wash it on a regular basis. Once, I was severely ill as a result of it because I had no experience of washing my body and clothes (XX7, male, 16 years old)*.

*I now take various precautions to protect myself after being exposed to diseases. I, for example, take frequent showers, but we still get sick so easily (XX4, male, 17 years old)*.

For some street children, the type of response to perceived or experienced health risks was based on experiential knowledge. A street child, for instance, disclosed that he tries to eat enough food, which he believes helps him cope with malaria:

*I sometimes keep myself safe to avoid disease. I have a recurrent malaria infection, so I'm susceptible to becoming ill if I don't get enough food, and it forces me to be in cold weather at one time and hot weather at another. As a result, I take great care to avoid recurrence of this disease, particularly by eating enough food (XX9, male, 14 years old)*.

Male street children who perceive that they are susceptible to risks of sexual violence, protecting themselves from potential perpetrators, especially strange street children, have been reported:

*If we find anyone who wants to have a sexual relationship with us, we will kill him. If something like that were to happen, no one would stay in this area. There are no girls either. We all came from the same area, so we're all too familiar with one another. Other mature boys have sexual relationships with girls, but we never allow them to sleep with us (XX2, 15, male)*.

Reports of reducing engagement in risky behaviors after realizing the negative consequences of such behaviors have also been reported.

*I smoke a little amount of cigarette. Many of us here share a single cigarette. We used to smoke two or three times a day, but now I only smoke once a day to avoid health problems. If I smoked a lot of cigarettes, it would even give me a headache. In addition, I will not sleep in public places where people urinate to avoid illness, but I can't do anything about the chilly weather (XX1, 15, Male)*.

The study also noted that research participants' preventive health behavior ranged from casual activities and efforts at the individual level to planned and organized activities of saving money and raising funds for emergency illness response. One street child reported:

*There is an older guy among us who is in charge of coordinating activities of collecting money regularly to deal with any health risks that our members may face. We have money that we have collected from our group members to use during emergencies such as when diseases occur or when people want to travel to their families. I, for example, have 700 birr deposited for same purpose (male, 15 years old)*.

In addition to preventive health behavior, we found that street children have a relatively high experience of healthcare-seeking behavior, which is expressed by responding to illness episodes. Whereas visits to healthcare facilities have been reported, purchasing drugs from nearby drug stores when feeling sick have also constituted aspects of health-seeking behavior:

…*I became severely ill yesterday after eating half-spoiled leftover food from hotels, but I was able to handle it after visiting a health center for treatment. I received free treatment at Zewditu hospital but was asked to pay 200 birr for medicines, which I did (XX4, male, 17 years old)*.

…*I go to the pharmacy to get medicine for any minor health issue (XX1, 15, Male)*.

…*For example, one of my old friends was so addicted that one day he got drunk, hit his head against the wall inside the train line, and was severely beaten by police. He lay on the same ground for 6 days without food, developing a swarm of louses on his clothing while no one noticed. We took him to the hospital on the sixth day, but he died immediately due to RF and severe bleeding from his head (XX7, male, 16 years). …There is also a child that I know who is currently undergoing treatment as a result of RF at a hospital that provides free medical care to us (XX7, male, 16 years)*.

Experiences of visiting healthcare facilities have been found not only in response to illness episodes but also that healthcare seeking behavior among girls of the street have also been reported in cases of maternity care services:

…*I faced no difficulties when I gave birth. It was after six months that I discovered my pregnancy status. I had regular pregnancy checkups until delivery. I was finally able to give birth at a health canter without any difficulties (XX6, female, 18 years)*.

It was indicated that the availability of free healthcare services played a role in promoting healthcare-seeking behavior among street children.

…*When a child becomes ill, we usually go to Yekatit 12 hospital and receive free treatment after waiting in line for a long time because no other health center treats us (XX3, male, 18)*.

*They go to Yekatit 12 hospital to receive free health care from a foreign woman who works there (XX6, 18, female)*.

## Discussion

The nature and extent of health-seeking behavior vary from one person to another or between groups, depending on different factors (Oberoi et al., 2016). A person's actions related to health behaviors promote good health and prevent health risks (Mushtaq et al., 2020). In this regard, studies (e.g., Eshita, 2018) show that street children are the most neglected part of society, with very limited access to health care and lack of awareness of available health services. According to Woan et al. (2013), street children's survival behaviors and exposure to poor shelters have resulted in morbidity, including infectious diseases, reproductive health, psychiatric diseases, and stunted growth. Although much has been said about the vulnerability of street children, little is known about their health-seeking behaviors. Hence, this study was undertaken to identify the health-seeking behavior of street children in Addis Ababa. To this end, both quantitative and qualitative data were collected from children on the street and analyzed to identify patterns in the data.

Perceived susceptibility refers to a person's perception that he or she may encounter the risks associated with [negative] health behavior, mostly embedded in the person's routine living experiences, which may expose him/her to a certain ill-health condition (Che et al., 2014). The results of the study indicated that most of the children and adolescents in the street perceive that they are susceptible to health risks, such as stomach ache, louse-borne diseases, headaches, illnesses caused by exposure to harsh weather, typhoid/typhus, and cardiac infection, as a result of the living conditions on the street. Disease risk perceptions are fundamental determinants of health behavior, and evidence regarding people's perceived susceptibility to a health threat is helpful for undertaking interventions that promote positive compliance (Harvey and Lawson, 2009; Ferrer and Klein, 2015). Accordingly, the majority of respondents had experience engaging in preventive health activities with the aim of protecting themselves from ill-health risks. Aspects of such preventive behavior include the experience of maintaining one's personal hygiene with a relatively high interval of taking a shower and performing physical exercises. According to Tarkang (2015), a person engages in preventive health behavior based on positive expectations that doing so would result in avoiding actual or perceived negative health conditions.

The other side of the finding about the health behavior of street children in the study area is their vulnerability to adverse health risks due to engaging in risky behaviors, such as smoking cigarettes, sniffing glue and benzene, unsafe sexual intercourse, and sharing personal materials with the potential to transmit diseases from one person to another. This finding is consistent with the results of a study conducted in Dhaka city (Eshita, 2018) which revealed that about half of street children engage in various risky behaviors such as smoking, unsafe sex, and abuse of drugs, which exposes them to physical and mental development problems. Furthermore, a study conducted in Northern Ethiopia (Brhane et al., 2014) revealed that one-third of street children had started sexual intercourse, over 60% of them had more than one sexual partner, and 40.6% had sexual intercourse with commercial sex workers. Moreover, the vulnerability of street children participating in this study was exacerbated by the absence of experience of visiting healthcare facilities for the purpose of undergoing general health check-ups among most of the respondents. Ali and de Muynck (2005) confirmed that street children are highly susceptible to adverse health outcomes such as physical injuries and respiratory and skin infections. Above all, Chowdhury et al. (2017) associated street children's exposure to various skin diseases and other communicable infections with their overall living conditions, such as overcrowded living situations, unhealthy sleeping areas, irregular baths, and fewer changes in clothes.

According to Adam and Aigbokhaode (2018), self-treatment is the most common type of illness response among people in developing countries, mainly because of the widely prevalent knowledge of traditional medical treatments and the use of alternative care services. In addition, Uche (2017) contended that people usually opt for the simplest form of treatment, which they deem more often than not, as the cheapest and most effective, and it is only when these simplest forms of treatment are adjudged as unsuccessful that the higher, more expensive, and conventional treatments are sought. In contrast, the results of the present study indicate that most respondents visited modern healthcare facilities whenever they felt sick, in addition to self-care practices and spiritual treatments. Moreover, this finding also differs from the results of a study conducted in Dhaka city by Eshita (2018) who concluded that street children often attempt self-medication, such as applying “masala” (spices) or “chuna” (quicklime) to wounds, drinking “soda” for gastro-intestinal problems, and taking over-the-counter drugs for all kinds of infections. Above all, Tegegne and Legese (2014) found that people who belong to the lowest social class in Addis Ababa, Ethiopia, do not visit professional care providers immediately after identifying disease symptoms and instead either ignore symptoms or seek other options. It is also indicated that, at the last stage, members of such social groups seek treatment from a trained allopathic.

For respondents who had never visited healthcare facilities, the most frequently mentioned reasons were financial problems, the disease being less severe, preference for self-care, fear of pain during medical injection, fear of long waiting hours in healthcare facilities, and lack of awareness. Similarly, Jalu et al. (2019) argued that low socio-demographic and economic status and poor exposure to health-related information were barriers to healthcare-seeking behavior. In addition, Eshita (2018) contended that the health-seeking behavior of street children is highly influenced by factors such as the availability of resources, knowledge of healthcare facilities, amount of waiting time to access healthcare services, travel distance to health centers, and faith in the healthcare provider. According to Akeju et al. (2016), high cost of medical care deters healthcare-seeking intentions. Moreover, similar to the results of the study, Andersen (1995) pointed out that enabling factors such as knowledge about how to access health services, income, health insurance, regular source of care, travel, and the extent and quality of social relationships affect individuals' healthcare-seeking behavior.

Kuuire et al. (2015) stated that although predisposing and enabling factors are necessary for healthcare utilization, they are not sufficient to lead to actual use, which is initiated by need that arises mainly as a result of the severity of illness. According to Oberoi et al. (2016), whether a person performs a particular health behavior is influenced by both the degree to which the disease is perceived by the person as threatening, and the extent to which a given health behavior is believed to be effective in terms of reducing negative health outcomes. Moreover, Andersen (1995) argued that people's healthcare-seeking behavior is determined by how they view their own general health and functional state, how they experience symptoms of illness, pain, and worries about their health, and whether or not they judge their problems to be of sufficient importance and magnitude to seek professional help. On the other hand, the results of binary logistic regression analysis between the outcome variable (respondents' tendency to visit healthcare facilities) and the independent variables (age, sex, education, residential background, religion, and duration on the street) have shown that respondents' healthcare-seeking behavior is significantly associated with the length of the period counted since they joined street life, while the other predictor variables were not significantly associated. This finding differs from most previous studies (e.g., Mackian, 2004; Siddiqui, 2014) in which the socio-demographic characteristics of respondents, such as age and sex, were significantly associated with healthcare-seeking behavior. It also differs from Andersen's (1995) predisposing factors that affect healthcare-seeking behavior, including sex, age, and educational status.

## Conclusions

Better prospects of health-seeking behavior, manifested both in the actions taken to prevent health risks and in positive compliance with healthcare use, have been found among children and adolescents of the street in Addis Ababa. Whereas street children perceive themselves to be susceptible to ill-health risks related to their living situations, responding to the perceived susceptibility mainly by maintaining personal hygiene and undertaking physical exercises have been identified. In addition, the experience of visiting healthcare facilities in response to illness symptoms has also constituted an aspect of street children's health-seeking behavior. Furthermore, while perceived severity of the disease, having an adequate amount of money, and access to free healthcare services promoted intentions for healthcare visits, financial problems, perceived less severity of illnesses, and preference for self-care were identified as barriers to healthcare seeking. On the other hand, in addition to their living conditions that have the potential to put street children at risk of exposure to various health problems, their vulnerability has been exacerbated by involvement in risky behaviors, such as smoking cigarettes, sniffing glue and benzene, and unprotected sexual practices. Efforts to create an inclusive society where marginalized sections of the urban community require children and adolescents of the street to be reintegrated into mainstream society. One way of doing this might be to create better access to modern healthcare services and empowering street children to maintain their health and wellbeing. Hence, state and non-state actors are expected to play key roles in this regard.

## Data availability statement

The original contributions presented in the study are included in the article/supplementary material, further inquiries can be directed to the corresponding author.

## Ethics statement

This study was approved by the Ethics Approval Committee of Dilla University. The research participants were first informed about the purpose of the research, including what role they expected from their side. Both verbal and written consent was obtained from all research participants. Additionally, a formal letter was obtained from the Department of Sociology at Addis Ababa University. This study was conducted in accordance with relevant guidelines.

## Author contributions

BZ conceived the research idea, took the leading role in data analysis, and prepared the manuscript. GS, KE, and GT participated in initiating the research idea, actively participated in the development of the research proposal, preparation of the data gathering instruments, data collection, data analysis, and preparation of the manuscript. All authors have read, edited, and approved the final manuscript.

## Footnotes

^1^RF stands for Relapsing Fever.
